# *trans*-Bis(4-amino­pyridine-κ*N*)bis­(quin­ox­aline-2,3-di­thiol­ato-κ^2^ *S*,*S*′)platinum(IV) dimethyl sulfoxide monosolvate

**DOI:** 10.1107/S2414314622001018

**Published:** 2022-02-03

**Authors:** Jiandu Hu, Volodymyr V. Nesterov, Bradley W. Smucker

**Affiliations:** a Austin College, 900 N Grand, Sherman, TX 75090, USA; b Department of Chemistry, University of North Texas, 1508 W. Mulberry, Denton, TX, 76201, USA; Vienna University of Technology, Austria

**Keywords:** crystal structure, platinum(IV), di­thiol­ene

## Abstract

In the structure of the title solvated complex, [Pt(C_8_H_4_N_2_S_2_)_2_(C_5_H_6_N_2_)_2_]·C_2_H_6_OS or *trans*-[Pt(4-*ap*)_2_(qdt)_2_]·dmso (4-*ap* = 4-amino­pyridyl, C_5_H_6_N_2_; qdt = quinoxaline-2,3-di­thiol­ate, C_8_H_4_N_2_S_2_; dmso = dimethyl sulfoxide, C_2_H_6_OS) the centrosymmetric complex exhibits Pt—S distances in agreement with other Pt^IV^—S bond lengths found in platinum(IV) di­thiol­ene complexes. The qdt ligands have inter­molecular inter­actions with an amine hydrogen atom on a 4-*ap* ligand (hydrogen bonding) and have sandwich π–π inter­actions with a neighboring qdt ligand.

## Abstract 2

The title compound exhibits typical Pt^IV^—S bond lengths. The packing of the complexes enable inter­molecular inter­actions between the qdt ligands (qdt = quinoxaline-2,3-di­thiol­ate) and an amine hydrogen atom (hydrogen bonding) or other qdt ligands (π–π stacking).

## Graphical abstract

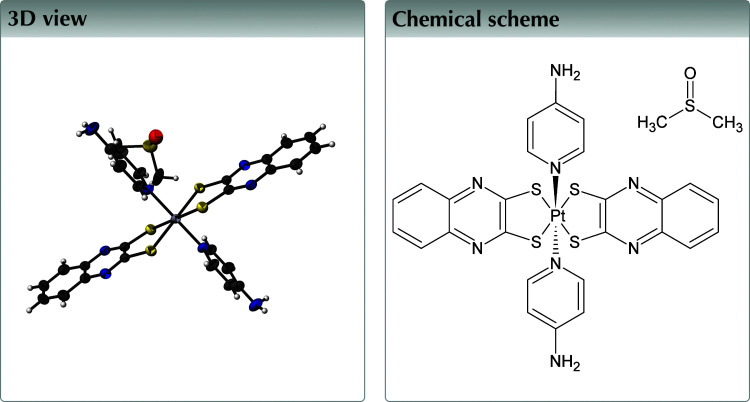

## Structure description

**Figure 1:**
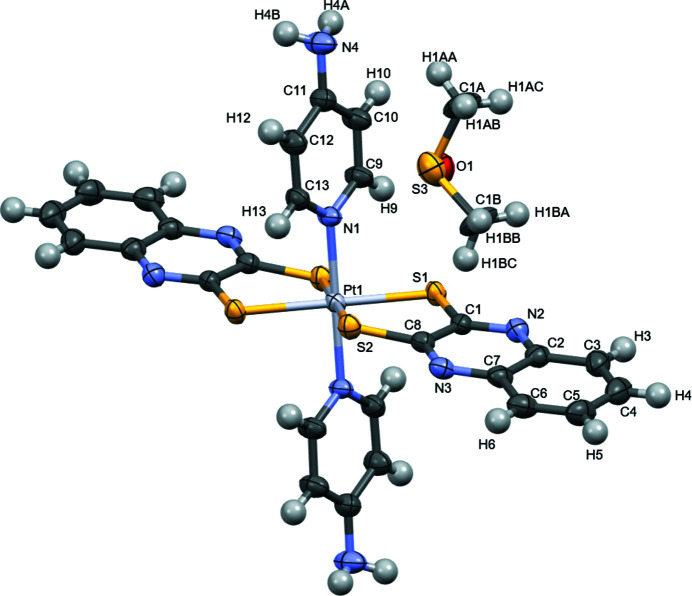
The mol­ecular structure of the title complex drawn with displacement ellipsoids at the 50% probability level. Non-labeled atoms are generated by symmetry operation −*x* + 1, −*y* + 1, −*z*. The disordered dmso solvate mol­ecule is shown with only one orientation.

The title *trans*-[Pt(4-*ap*)_2_(qdt)_2_] ((4-*ap* = 4-amino­pyridyl; qdt = quinoxaline-2,3-di­thiol­ate) complex is located about an inversion center and has the central Pt^IV^ atom in a pseudo-octa­hedral N_2_S_4_ coordination environment (Fig. 1 ▸). In contrast to the shorter Pt^II^—S distances in salts of [Pt(mnt)_2_]^2–^ (mnt = maleo­nitrile­dithiol­ate**)**, such as 2.295 (2) and 2.2958 (19) Å with the tetra­phenyl­phosphine cation (Begum *et al.*, 2014 ▸) or 2.290 (2) and 2.282 (2) Å with the tetra­butyl­ammonium cation (Güntner *et al.*, 1989 ▸), the Pt^IV^—S distances of the title coordination compound are 2.3514 (11) Å (Pt1—S1) and 2.3495 (11) Å (Pt1—S2). These distances are similar to those in other platinum(IV) complexes containing bis­(di­thiol­ene) ligands and either *trans*-bis­(NH_3_) co-ligands, with Pt—S distances of 2.3434 (8) and 2.3461 (7) Å (Siddiqui *et al.*, 2020 ▸), or *trans*-bis­(PMe_3_) co-ligands, with a Pt—S distance of 2.3619 (8) Å (Chandrasekaran *et al.*, 2014 ▸). The Pt1—N1 distance in the title complex is 2.063 (4) Å, which is similar to the Pt—N distance of 2.055 (2) Å in the aforementioned *trans*-[Pt(NH_3_)_2_(mnt)_2_] complex (Siddiqui *et al.*, 2020 ▸).

**Figure 2:**
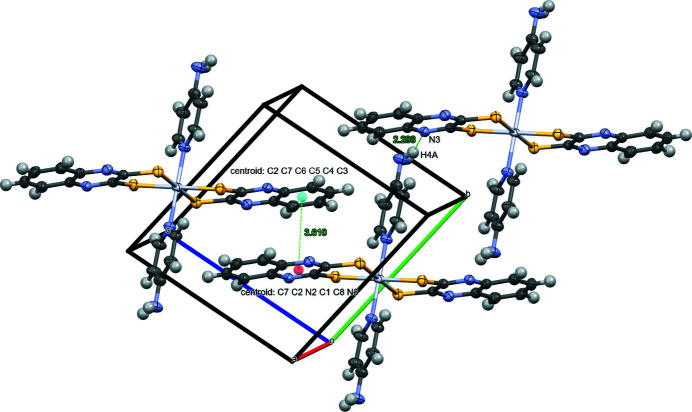
The packing of the complexes showing the hydrogen bonding between the H4*A* amine hydrogen atom and the N3 (*x*, *y* + 1, *z*) atom on a neighboring qdt ligand as well as the sandwich orientation between adjacent qdt ligands and the distance (Å) between centroids of two qdt rings. Displacement ellipsoids are drawn at the 50% probability level; the dmso solvate is omitted for clarity.

The chelating qdt ligands of this platinum(IV) complex are slightly canted relative to the platinum-sulfur atoms, with a 15.59 (11)° angle between the plane of all the non-H atoms of the qdt ligand *versus* the plane containing Pt, S1, S2, S1 (1 − *x*, 1 − *y*, −*z*) and S2 (1 − *x*, 1 − *y*, −*z*). This tilt enables sandwich packing between inter­molecular qdt ligands with a distance between centroids of the two qdt rings of 3.610 Å (Fig. 2 ▸), within the range of π–π inter­actions (Sinnokrot *et al.*, 2002 ▸). The basicity of the nitro­gen atom on the coordinating qdt ligand (Cummings & Eisenberg, 1995*b* ▸) makes it suitable for hydrogen bonding. This is observed between the amine hydrogen H4*A* and the N3 (*x*, *y* + 1, *z*) atom on a neighboring qdt ligand, with a distance of 2.23 Å (Table 1 ▸, Fig. 2 ▸). N—H⋯O hydrogen bonding is observed between the complex and the O atom of the dmso solvent mol­ecule.

## Synthesis and crystallization

An orange solution of the anionic qdt ligand was prepared by combining 9.3 mg of 2,3-quinoxalinedi­thiol (Cummings & Eisenberg, 1995*a* ▸) and 7.7 mg of NaHCO_3_ with 25 ml of water and heating at 333 K for 5 h. Upon cooling to room temperature, the orange solution was added, *via* cannula, to a Schlenk flask containing 34.3 mg of [Pt(4-*ap*)_4_](BF_4_)_2_, prepared in a similar manner to [Pt(pyz)_4_](BF_4_)_2_ (Derry *et al.*, 2008 ▸), and 7.9 mg of NaHCO_3_. The solution was stirred for 7 d with the exclusion of light. The resulting orange–brown solid was collected *via* vacuum filtration in air and washed with 3 × 10 ml of water and 15 ml of diethyl ether to give 7.4 mg (28% for [Pt(4-*ap*)_2_(qdt)]). Oxidation of platinum(II) to platinum(IV) likely occurred upon prolonged air exposure of the compound in solution (Geiger *et al.*, 2001 ▸; Siddiqui *et al.*, 2020 ▸).

Light-yellow crystals of the title compound were grown by slow diffusion of water into a dmso solution of the platinum complex.

## Refinement

Crystal data, data collection and structure refinement details are summarized in Table 2 ▸. The dmso solvent mol­ecule is disordered about an inversion center and shows half occupancy.

## Supplementary Material

Crystal structure: contains datablock(s) I. DOI: 10.1107/S2414314622001018/wm4160sup1.cif

Structure factors: contains datablock(s) I. DOI: 10.1107/S2414314622001018/wm4160Isup2.hkl

Supporting information file. DOI: 10.1107/S2414314622001018/wm4160Isup3.mol

CCDC reference: 2145270

Additional supporting information:  crystallographic information; 3D view; checkCIF report

## Figures and Tables

**Table 1:** Hydrogen-bond geometry (Å, °)

*D*—H⋯*A*	*D*—H	H⋯*A*	*D*⋯*A*	*D*—H⋯*A*
N4—H4*A*⋯N3^i^	0.87	2.30	3.085 (7)	151
N4—H4*B*⋯O1^ii^	0.87	2.28	3.045 (11)	148

Symmetry codes: (i) 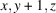 ; (ii) 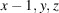 .

**Table 2:** Experimental details

Crystal data	
Chemical formula	[Pt(C_8_H_4_N_2_S_2_)_2_(C_5_H_6_N_2_)_2_]·C_2_H_6_OS
*M*_r_	845.96
Crystal system, space group	Triclinic, *P* 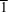
Temperature (K)	200
*a*, *b*, *c* (Å)	7.74108 (18), 9.8690 (2), 10.47021 (18)
α, β, γ (°)	99.6963 (16), 102.9798 (17), 100.9394 (19)
*V* (Å^3^)	746.43 (3)
*Z*	1
Radiation type	Cu *K*α
μ (mm^−1^)	12.39
Crystal size (mm)	0.03 × 0.02 × 0.01

Data collection	
Diffractometer	XtaLAB Synergy, Dualflex, HyPix
Absorption correction	Multi-scan (*CrysAlis PRO*; Rigaku OD, 2019 ▸)
*T*_min_, *T*_max_	0.671, 1.000
No. of measured, independent and observed [*I* > 2σ(*I*)] reflections	15557, 3130, 3097
*R*_int_	0.046
(sin θ/λ)_max_ (Å^−1^)	0.634

Refinement	
*R*[*F* ^2^ > 2σ(*F* ^2^)], *wR*(*F* ^2^), *S*	0.033, 0.083, 1.11
No. of reflections	3130
No. of parameters	218
H-atom treatment	H-atom parameters constrained
Δρ_max_, Δρ_min_ (e Å^−3^)	1.33, −1.21

Computer programs: *CrysAlis PRO* (Rigaku OD, 2019 ▸), *SHELXL* (Sheldrick, 2015 ▸), *OLEX2* (Dolomanov *et al.*, 2009 ▸) *Mercury* (Macrae *et al.*, 2020 ▸), and *OLEX2* (Dolomanov *et al.*, 2009 ▸).

## full crystallographic data

### Crystal data

**Table:**

[Pt(C_8_H_4_N_2_S_2_)_2_(C_5_H_6_N_2_)_2_]·C_2_H_6_OS	*Z* = 1
*M**_r_* = 845.96	*F*(000) = 416
Triclinic, *P*1	*D*_x_ = 1.882 Mg m^−^^3^
*a* = 7.74108 (18) Å	Cu *K*α radiation, λ = 1.54184 Å
*b* = 9.8690 (2) Å	Cell parameters from 10134 reflections
*c* = 10.47021 (18) Å	θ = 4.7–77.2°
α = 99.6963 (16)°	µ = 12.39 mm^−^^1^
β = 102.9798 (17)°	*T* = 200 K
γ = 100.9394 (19)°	Plate, clear light yellow
*V* = 746.43 (3) Å^3^	0.03 × 0.02 × 0.01 mm

### Data collection

**Table:**

XtaLAB Synergy, Dualflex, HyPix diffractometer	3130 independent reflections
Radiation source: micro-focus sealed X-ray tube, PhotonJet (Cu) X-ray Source	3097 reflections with *I* > 2σ(*I*)
Mirror monochromator	*R*_int_ = 0.046
Detector resolution: 10.0000 pixels mm^-1^	θ_max_ = 77.7°, θ_min_ = 4.4°
ω scans	*h* = −9→9
Absorption correction: multi-scan (CrysAlisPro; Rigaku OD, 2019)	*k* = −12→12
*T*_min_ = 0.671, *T*_max_ = 1.000	*l* = −11→13
15557 measured reflections	

### Refinement

**Table:**

Refinement on *F*^2^	Hydrogen site location: mixed
Least-squares matrix: full	H-atom parameters constrained
*R*[*F*^2^ > 2σ(*F*^2^)] = 0.033	*w* = 1/[σ^2^(*F*_o_^2^) + (0.0335*P*)^2^ + 2.9739*P*] where *P* = (*F*_o_^2^ + 2*F*_c_^2^)/3
*wR*(*F*^2^) = 0.083	(Δ/σ)_max_ < 0.001
*S* = 1.11	Δρ_max_ = 1.33 e Å^−^^3^
3130 reflections	Δρ_min_ = −1.21 e Å^−^^3^
218 parameters	Extinction correction: SHELXL2018/3 (Sheldrick 2015), Fc^*^=kFc[1+0.001xFc^2^λ^3^/sin(2θ)]^-1/4^
0 restraints	Extinction coefficient: 0.00059 (15)

### Special details

**Table:**

Geometry. All esds (except the esd in the dihedral angle between two l.s. planes) are estimated using the full covariance matrix. The cell esds are taken into account individually in the estimation of esds in distances, angles and torsion angles; correlations between esds in cell parameters are only used when they are defined by crystal symmetry. An approximate (isotropic) treatment of cell esds is used for estimating esds involving l.s. planes.

### Fractional atomic coordinates and isotropic or equivalent isotropic displacement parameters (Å^2^)

**Table:**

	*x*	*y*	*z*	*U*_iso_*/*U*_eq_	Occ. (<1)
Pt1	0.500000	0.500000	0.000000	0.02644 (11)	
S1	0.81596 (15)	0.56972 (12)	0.10110 (12)	0.0312 (2)	
S2	0.46861 (16)	0.38023 (12)	0.17238 (12)	0.0317 (2)	
N1	0.4661 (5)	0.6809 (4)	0.1128 (4)	0.0302 (8)	
N2	1.0148 (6)	0.4701 (4)	0.2823 (4)	0.0340 (9)	
N3	0.7159 (6)	0.2878 (4)	0.3258 (4)	0.0326 (9)	
N4	0.4058 (8)	1.0482 (5)	0.3409 (5)	0.0487 (12)	
H4A	0.459240	1.121162	0.316472	0.058*	
H4B	0.291608	1.048543	0.329940	0.058*	
C1	0.8464 (6)	0.4641 (5)	0.2183 (5)	0.0286 (9)	
C2	1.0401 (7)	0.3838 (5)	0.3712 (5)	0.0344 (10)	
C3	1.2200 (8)	0.3870 (6)	0.4442 (6)	0.0429 (12)	
H3	1.319219	0.449873	0.434033	0.051*	
C4	1.2469 (9)	0.2974 (7)	0.5296 (6)	0.0485 (14)	
H4	1.364634	0.300343	0.577882	0.058*	
C5	1.0979 (9)	0.2013 (6)	0.5445 (6)	0.0475 (14)	
H5	1.117997	0.140023	0.601820	0.057*	
C6	0.9238 (9)	0.1967 (6)	0.4759 (5)	0.0441 (13)	
H6	0.826180	0.132057	0.486098	0.053*	
C7	0.8918 (7)	0.2901 (5)	0.3894 (5)	0.0344 (10)	
C8	0.6925 (7)	0.3742 (5)	0.2431 (5)	0.0297 (9)	
C9	0.5791 (8)	0.8073 (5)	0.1265 (6)	0.0392 (12)	
H9	0.672540	0.811304	0.083782	0.047*	
C10	0.5625 (8)	0.9305 (6)	0.2007 (6)	0.0419 (12)	
H10	0.643536	1.015621	0.207214	0.050*	
C11	0.4242 (8)	0.9282 (5)	0.2661 (5)	0.0377 (11)	
C12	0.3087 (8)	0.7949 (6)	0.2519 (6)	0.0408 (12)	
H12	0.214927	0.787183	0.294259	0.049*	
C13	0.3334 (7)	0.6769 (5)	0.1764 (5)	0.0344 (10)	
H13	0.255025	0.590151	0.168660	0.041*	
S3	1.0758 (5)	1.0293 (4)	0.0794 (4)	0.0557 (8)	0.5
O1	1.0037 (13)	0.9818 (10)	0.1907 (10)	0.058 (2)	0.5
C1A	1.000 (3)	0.8879 (17)	−0.058 (2)	0.072 (5)	0.5
H1AA	1.061993	0.814997	−0.040118	0.108*	0.5
H1AB	1.023516	0.918671	−0.135433	0.108*	0.5
H1AC	0.870658	0.851496	−0.073236	0.108*	0.5
C1B	0.950 (3)	1.150 (2)	0.030 (2)	0.072 (5)	0.5
H1BA	0.821992	1.107133	0.008588	0.108*	0.5
H1BB	0.979222	1.177789	−0.047899	0.108*	0.5
H1BC	0.979797	1.232401	0.101768	0.108*	0.5

### Atomic displacement parameters (Å^2^)

**Table:**

	*U*^11^	*U*^22^	*U*^33^	*U*^12^	*U*^13^	*U*^23^
Pt1	0.02549 (16)	0.02164 (15)	0.02864 (16)	0.00156 (10)	0.00498 (10)	0.00351 (10)
S1	0.0253 (5)	0.0292 (5)	0.0350 (6)	−0.0001 (4)	0.0036 (4)	0.0095 (4)
S2	0.0294 (6)	0.0311 (6)	0.0341 (6)	0.0030 (4)	0.0081 (5)	0.0111 (5)
N1	0.032 (2)	0.0230 (18)	0.035 (2)	0.0056 (15)	0.0114 (17)	0.0015 (15)
N2	0.033 (2)	0.033 (2)	0.033 (2)	0.0061 (17)	0.0046 (17)	0.0048 (17)
N3	0.040 (2)	0.0248 (19)	0.030 (2)	0.0051 (16)	0.0079 (17)	0.0045 (16)
N4	0.055 (3)	0.031 (2)	0.060 (3)	0.008 (2)	0.025 (3)	0.000 (2)
C1	0.029 (2)	0.025 (2)	0.029 (2)	0.0064 (17)	0.0039 (18)	0.0018 (17)
C2	0.041 (3)	0.033 (2)	0.029 (2)	0.012 (2)	0.006 (2)	0.0035 (19)
C3	0.040 (3)	0.047 (3)	0.036 (3)	0.014 (2)	0.003 (2)	0.002 (2)
C4	0.055 (4)	0.054 (3)	0.030 (3)	0.025 (3)	−0.004 (2)	0.001 (2)
C5	0.066 (4)	0.041 (3)	0.033 (3)	0.022 (3)	−0.001 (3)	0.009 (2)
C6	0.062 (4)	0.033 (3)	0.034 (3)	0.012 (2)	0.007 (3)	0.006 (2)
C7	0.044 (3)	0.029 (2)	0.026 (2)	0.010 (2)	0.003 (2)	0.0031 (18)
C8	0.034 (2)	0.025 (2)	0.028 (2)	0.0053 (18)	0.0069 (19)	0.0029 (18)
C9	0.040 (3)	0.025 (2)	0.052 (3)	0.001 (2)	0.022 (2)	0.003 (2)
C10	0.045 (3)	0.028 (2)	0.051 (3)	0.001 (2)	0.020 (3)	0.001 (2)
C11	0.040 (3)	0.031 (2)	0.040 (3)	0.008 (2)	0.010 (2)	0.004 (2)
C12	0.038 (3)	0.035 (3)	0.049 (3)	0.003 (2)	0.018 (2)	0.005 (2)
C13	0.032 (2)	0.028 (2)	0.041 (3)	0.0019 (19)	0.011 (2)	0.004 (2)
S3	0.0454 (16)	0.0553 (18)	0.0601 (19)	−0.0016 (14)	0.0131 (14)	0.0125 (15)
O1	0.053 (5)	0.062 (6)	0.065 (6)	0.013 (4)	0.023 (4)	0.020 (5)
C1A	0.078 (13)	0.045 (9)	0.108 (16)	0.036 (8)	0.035 (11)	0.020 (9)
C1B	0.087 (13)	0.063 (11)	0.087 (12)	0.055 (10)	0.026 (10)	0.026 (9)

### Geometric parameters (Å, º)

**Table:**

Pt1—S1	2.3514 (11)	C4—C5	1.404 (10)
Pt1—S1^i^	2.3514 (11)	C5—H5	0.9300
Pt1—S2	2.3495 (11)	C5—C6	1.366 (9)
Pt1—S2^i^	2.3495 (11)	C6—H6	0.9300
Pt1—N1	2.063 (4)	C6—C7	1.413 (7)
Pt1—N1^i^	2.063 (4)	C9—H9	0.9300
S1—C1	1.743 (5)	C9—C10	1.373 (7)
S2—C8	1.741 (5)	C10—H10	0.9300
N1—C9	1.346 (6)	C10—C11	1.393 (8)
N1—C13	1.342 (6)	C11—C12	1.407 (7)
N2—C1	1.310 (6)	C12—H12	0.9300
N2—C2	1.371 (7)	C12—C13	1.363 (7)
N3—C7	1.369 (7)	C13—H13	0.9300
N3—C8	1.323 (6)	S3—O1	1.505 (10)
N4—H4A	0.8662	S3—C1A	1.728 (19)
N4—H4B	0.8665	S3—C1B	1.752 (16)
N4—C11	1.355 (7)	C1A—H1AA	0.9600
C1—C8	1.446 (7)	C1A—H1AB	0.9600
C2—C3	1.421 (8)	C1A—H1AC	0.9600
C2—C7	1.402 (8)	C1B—H1BA	0.9600
C3—H3	0.9300	C1B—H1BB	0.9600
C3—C4	1.370 (8)	C1B—H1BC	0.9600
C4—H4	0.9300		
			
S1—Pt1—S1^i^	180.0	C5—C6—H6	120.0
S2—Pt1—S1	88.43 (4)	C5—C6—C7	120.0 (6)
S2^i^—Pt1—S1^i^	88.43 (4)	C7—C6—H6	120.0
S2—Pt1—S1^i^	91.57 (4)	N3—C7—C2	121.3 (4)
S2^i^—Pt1—S1	91.57 (4)	N3—C7—C6	119.1 (5)
S2^i^—Pt1—S2	180.0	C2—C7—C6	119.6 (5)
N1^i^—Pt1—S1^i^	89.99 (12)	N3—C8—S2	116.7 (4)
N1^i^—Pt1—S1	90.01 (12)	N3—C8—C1	121.3 (4)
N1—Pt1—S1	89.99 (12)	C1—C8—S2	121.9 (4)
N1—Pt1—S1^i^	90.01 (12)	N1—C9—H9	118.6
N1—Pt1—S2	90.33 (12)	N1—C9—C10	122.9 (5)
N1^i^—Pt1—S2^i^	90.33 (12)	C10—C9—H9	118.6
N1—Pt1—S2^i^	89.67 (12)	C9—C10—H10	120.0
N1^i^—Pt1—S2	89.67 (12)	C9—C10—C11	120.0 (5)
N1^i^—Pt1—N1	180.0	C11—C10—H10	120.0
C1—S1—Pt1	103.03 (16)	N4—C11—C10	121.2 (5)
C8—S2—Pt1	102.34 (17)	N4—C11—C12	122.6 (5)
C9—N1—Pt1	120.7 (3)	C10—C11—C12	116.2 (5)
C13—N1—Pt1	121.6 (3)	C11—C12—H12	119.8
C13—N1—C9	117.7 (4)	C13—C12—C11	120.5 (5)
C1—N2—C2	117.3 (4)	C13—C12—H12	119.8
C8—N3—C7	117.1 (4)	N1—C13—C12	122.7 (5)
H4A—N4—H4B	108.6	N1—C13—H13	118.7
C11—N4—H4A	109.7	C12—C13—H13	118.7
C11—N4—H4B	110.6	O1—S3—C1A	106.8 (8)
N2—C1—S1	116.9 (4)	O1—S3—C1B	104.6 (9)
N2—C1—C8	121.8 (4)	C1A—S3—C1B	103.1 (11)
C8—C1—S1	121.3 (4)	S3—C1A—H1AA	109.5
N2—C2—C3	119.6 (5)	S3—C1A—H1AB	109.5
N2—C2—C7	121.1 (5)	S3—C1A—H1AC	109.5
C7—C2—C3	119.3 (5)	H1AA—C1A—H1AB	109.5
C2—C3—H3	120.0	H1AA—C1A—H1AC	109.5
C4—C3—C2	120.0 (6)	H1AB—C1A—H1AC	109.5
C4—C3—H3	120.0	S3—C1B—H1BA	109.5
C3—C4—H4	119.8	S3—C1B—H1BB	109.5
C3—C4—C5	120.4 (6)	S3—C1B—H1BC	109.5
C5—C4—H4	119.8	H1BA—C1B—H1BB	109.5
C4—C5—H5	119.6	H1BA—C1B—H1BC	109.5
C6—C5—C4	120.7 (5)	H1BB—C1B—H1BC	109.5
C6—C5—H5	119.6		
			
Pt1—S1—C1—N2	173.6 (3)	C2—C3—C4—C5	0.6 (8)
Pt1—S1—C1—C8	−6.3 (4)	C3—C2—C7—N3	177.0 (5)
Pt1—S2—C8—N3	−166.6 (3)	C3—C2—C7—C6	−2.5 (7)
Pt1—S2—C8—C1	16.4 (4)	C3—C4—C5—C6	−0.9 (9)
Pt1—N1—C9—C10	−179.9 (5)	C4—C5—C6—C7	−0.5 (9)
Pt1—N1—C13—C12	179.8 (4)	C5—C6—C7—N3	−177.3 (5)
S1—C1—C8—S2	−7.3 (6)	C5—C6—C7—C2	2.2 (8)
S1—C1—C8—N3	175.8 (4)	C7—N3—C8—S2	−175.4 (3)
N1—C9—C10—C11	0.1 (10)	C7—N3—C8—C1	1.7 (7)
N2—C1—C8—S2	172.8 (4)	C7—C2—C3—C4	1.1 (8)
N2—C1—C8—N3	−4.1 (7)	C8—N3—C7—C2	2.3 (7)
N2—C2—C3—C4	−177.7 (5)	C8—N3—C7—C6	−178.2 (5)
N2—C2—C7—N3	−4.2 (7)	C9—N1—C13—C12	0.7 (8)
N2—C2—C7—C6	176.3 (5)	C9—C10—C11—N4	179.4 (6)
N4—C11—C12—C13	−179.5 (6)	C9—C10—C11—C12	0.6 (9)
C1—N2—C2—C3	−179.4 (4)	C10—C11—C12—C13	−0.7 (9)
C1—N2—C2—C7	1.8 (7)	C11—C12—C13—N1	0.0 (9)
C2—N2—C1—S1	−177.8 (3)	C13—N1—C9—C10	−0.7 (8)
C2—N2—C1—C8	2.2 (7)		

Symmetry code: (i) −*x*+1, −*y*+1, −*z*.

### Hydrogen-bond geometry (Å, º)

**Table:**

*D*—H···*A*	*D*—H	H···*A*	*D*···*A*	*D*—H···*A*
N4—H4*A*···N3^ii^	0.87	2.30	3.085 (7)	151
N4—H4*B*···O1^iii^	0.87	2.28	3.045 (11)	148

Symmetry codes: (ii) *x*, *y*+1, *z*; (iii) *x*−1, *y*, *z*.
